# The Diagnosis of Minimal Change Disease in Diabetic Nephropathy

**DOI:** 10.1100/tsw.2005.106

**Published:** 2005-09-29

**Authors:** M. Barry Stokes

**Affiliations:** Department of Pathology, Renal Pathology Laboratory, VC14-224, Columbia University College of Physicians and Surgeons, New York, NY 10032, USA

**Keywords:** diabetes, nephrotic syndrome, minimal change disease

## Abstract

Proteinuria is common in diabetic patients and usually reflects the presence of diabetic glomerulosclerosis. This paper reviews the differential diagnosis of proteinuria in diabetic patients and discusses the role of renal biopsy examination in identification and management of minimal change disease in this cohort. Identification of nondiabetic glomerular disease requires careful correlation of clinical history and renal biopsy findings and may have important implications for prognosis and therapy.

## INTRODUCTION

Diabetes is the single leading cause of end-stage renal disease in the U.S. and Western Europe. The cumulative incidence of kidney disease in both type I and type II diabetes mellitus is approximately 25% after 20 years. The natural history of diabetic nephropathy is best described for type I diabetes where the onset of disease is more clearly defined[1]. Following a clinically silent phase of 5–10 years, in which there may be elevated glomerular filtration rate and glomerular hypertrophy, patients develop microalbuminuria (30–300 mg/day) accompanied by normal renal function. Glomerular findings at this stage range from near normal with minimal mesangial expansion, to mild diabetic glomerulosclerosis[2]. Renal biopsies of type 2 diabetics with microalbuminuria may show severe tubulointerstitial, vascular, or glomerulosclerosis attributable to aging and/or hypertension[2]. Over time, a subset of microalbuminuric patients develops overt diabetic nephropathy, characterized by increasing proteinuria, hypertension, and progressive renal failure. The pathologic findings consist of progressively more severe diffuse or nodular glomerulosclerosis. In most cases of diabetic nephropathy, renal function declines inexorably and progresses to end-stage renal disease, although some patients have shown remission of proteinuria and stabilization of renal function with ACE inhibitor therapy[3].

A subset of patients with diabetic nephropathy develop nephrotic syndrome, characterized by urinary protein excretion greater than 3.5 g/day, hypoalbuminemia, hypercholesterolemia, and peripheral edema usually after a long period of subnephrotic proteinuria and accompanied by progressive renal failure. The onset of nephrotic syndrome in diabetic subjects without a prodrome of subnephrotic proteinuria suggests the possibility of a superimposed nondiabetic glomerular disease.

## NONDIABETIC GLOMERULAR DISEASE IN DIABETIC PATIENTS

A wide spectrum of nondiabetic glomerular disease has been described in diabetic patients[4,5,6,7]. The frequency of nondiabetic glomerular diseases, either coexistent with or superimposed on diabetic glomerulosclerosis, is difficult to ascertain, as these reflect regional differences in renal biopsy selection criteria, as well as the influence of geographic and ethnic factors that influence the incidence of glomerular disease. In a relatively homogenous study of 393 Northern Italian patients with type 2 diabetes, the most common glomerular diseases other than diabetic glomerulosclerosis were membranous nephropathy (10%), acute postinfectious glomerulonephritis (9%), and IgA nephropathy (9%). In the same study, 22 cases (6%) were classified as either minimal change disease (MCD) or focal segmental glomerulosclerosis, but the precise clinical and pathologic characteristics of these individuals were not detailed[5]. None of the cases of MCD/focal segmental glomerulosclerosis showed evidence of coexistent diabetic glomerulosclerosis. Another study reviewed the renal biopsy findings in 16 Indian patients with type 2 diabetes who lacked evidence of retinopathy and who were selected for renal biopsy because of urinary protein excretion greater than 1 g/day[8]. Eight patients (50%) had features of diabetic nephropathy, five (33.3%) had membranous nephropathy, two (12.5%) had minimal change nephropathy, whereas one biopsy (6.2%) showed tubulointerstitial disease[8].

## DIAGNOSIS OF MINIMAL CHANGE DISEASE IN DIABETIC PATIENTS

MCD is a major cause of idiopathic nephrotic syndrome in both children and adults. Typically, MCD presents with sudden or “explosive” onset of full nephrotic syndrome, i.e., peripheral edema, heavy proteinuria, hypoalbuminemia, and hypercholesterolemia, developing over a period of days, not weeks or months. In most individuals with MCD, the nephrotic syndrome is exquisitely steroid sensitive. Reduced glomerular filtration rate is not uncommon in MCD and a subset of patients present with acute renal failure and nephrotic syndrome[9]. However, progression to end-stage renal disease is extremely rare. MCD is characterized pathologically by glomeruli that appear normal by light microscopy and show diffuse effacement of visceral epithelial cell (podocyte) foot processes by electron microscopy (Fig. 1). Rarely, MCD may coexist with an unrelated primary glomerular disease, such as IgA nephropathy[10], HIV infection[11], or systemic lupus erythematosus[12] in which case other pathologic features related to these conditions may also be encountered in the renal biopsy. By convention, a diagnosis of MCD is excluded in cases where there are other pathologic findings that would account for the nephrotic syndrome, such as subepithelial immune complex deposits (membranous nephropathy) or amyloid deposits.

MCD is a well-described, albeit uncommon, cause of nephrotic syndrome in diabetic patients. In a review of eight published renal biopsy series totaling 533 patients with type 2 diabetes, Olsen and Mogensen[4] identified 20 cases (3.7%) that were described as having MCD or “minimal lesion”. At least 22 cases have been described with sufficient clinical and/or pathologic data to support a diagnosis of MCD in the setting of diabetes (summarized in Table 1)[13,14,15,16,17,18,19,20,21,22,23,24,25]. The majority of these patients were children (median age 15 years, range 2 months to 67 years) in whom the nephrotic syndrome presented simultaneously with or shortly after the diagnosis of insulin-dependent type 1 diabetes mellitus. Two case reports documented MCD occurring in the setting of adult-onset type 2 diabetes mellitus[21,25]. In two cases, MCD preceded the diagnosis of diabetes mellitus and only four patients had diabetes of 10 or more years’ duration when the nephrotic syndrome appeared. In all cases, there was prompt resolution of proteinuria, either spontaneously or following steroid or other immunosuppressive therapy. In this predominantly pediatric cohort, renal function was usually preserved and all five cases with renal impairment at presentation showed improved renal function following remission of the nephrotic syndrome. One patient had reversible acute renal failure that was likely related to hemodynamic perturbation in the setting of nephrotic syndrome[25]. Progressive renal failure occurred in only one individual, a woman with an 18-year history of diabetes and relapsing minimal change who developed progressively more severe diabetic glomerulosclerosis on repeat renal biopsies[18].

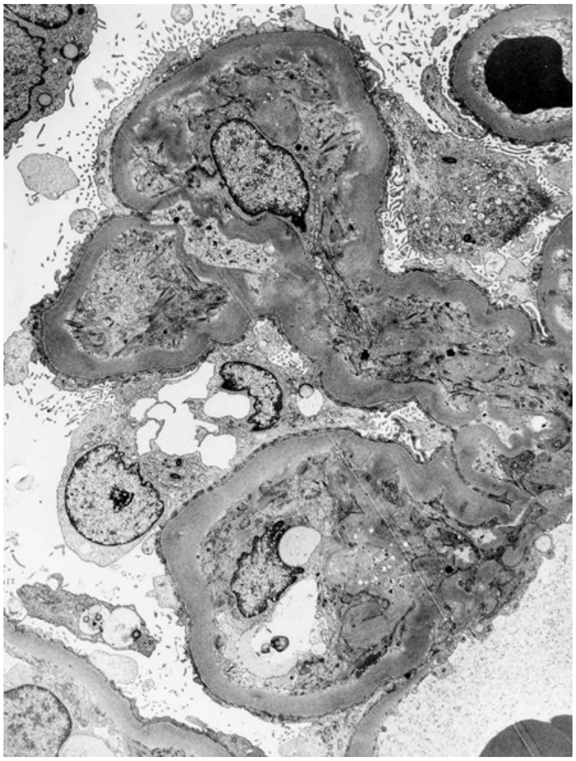

**FIGURE 1.** Electron microscopy showing diffuse effacement of podocyte foot processes with microvillous surface projections, consistent with MCD. Note also that underlying glomerular basement membranes are thickened, consistent with early diabetic nephropathy. (Electron micrograph, original magnification × 2,000.)

In most cases of MCD in diabetic subjects, renal biopsy shows either no significant histologic alterations[14,19,20,22,23] or only mild mesangial sclerosis and glomerular basement membrane thickening, consistent with early diabetic nephropathy[14,16,18,21,23,25] (Fig. 2). In all of these cases, foot process effacement was usually described as extensive (Fig. 1). The diagnosis of MCD in the setting of nodular diabetic glomerulosclerosis is problematic as diffuse foot process effacement and nephrotic proteinuria are common to both of these conditions. Two reported cases showed features of well-established diabetic nephropathy, one with diffuse glomerulosclerosis[22] and one with nodular glomerulosclerosis[15]. Although the extent of foot process effacement in these two cases was not described, the spontaneous complete remission of nephrotic syndrome (neither having received corticosteroids) supported the interpretation of superimposed MCD. In the setting of a renal biopsy showing nodular diabetic glomerulosclerosis, a diagnosis of MCD should only be considered where there has been abrupt onset of nephrotic syndrome and a documented absence of proteinuria in the recent past; clearly, such cases are exceedingly rare.

The occurrence of renal failure in the setting of diabetic nephropathy is usually a harbinger of end-stage renal disease. Exceptions include superimposed hemodynamic insults that compromise renal perfusion, such as in the setting of pregnancy where fluid shifts may lead to reversible acute renal failure[26]. The occurrence of acute renal failure in the setting of MCD, on the other hand, is a common reversible phenomenon, most frequently encountered in older patients who have significant underlying arteriosclerosis[27]. Investigators have described a high frequency of tubular simplification and interstitial edema consistent with acute tubular necrosis in this condition[9,27]. The pathogenesis of the acute renal failure in the setting of anasarca is poorly understood and has been attributed to hemodynamic

**TABLE 1**
**Summary of Reported Cases of Diabetes Mellitus and Coexistent MCD**

| Case # | Ref. # | Age (Years) at Onset of DM | Age (Years) at Onset of NS | UP (g/day) | Alb (g/dL) | ARF | Cr (mg/dL) | Renal Biopsy Findings | Therapy | Outcome |
|---|---|---|---|---|---|---|---|---|---|---|
| 1 | [13] | 8 | 8 | 3.3 | 1.6 | N | | n.d. | CS | Remiss. |
| 2 | [14] | 4.5 | 4.5+1m | 3.4 | 1.2 | N | | MCD | None | Remiss. |
| 3 | [14] | 8 | 8+1m | 7.2 | 1.5 | Y | 2.2 | MCD | CS,CP | Remiss. |
| 4 | [14] | 3 | 3+11m | 14 | 0.4 | N | | MCD | CS | Remiss. |
| 5 | [14] | 5 | 8 | 17 | 1.4 | N | | MCD DGS+MCD | CS | Remiss. |
| 6 | [14] | 2 mo | 10 | 7.3 | 1.1 | N | | | CS | Remiss. |
| 7 | [15] | 18 | 28 | 9.6 | 1.3 | Y | 1.6 | DGS | Diuretics | Remiss. |
| 8 | [16] | 23 | 38 | 7 | 1.2 | N | | DGS+MCD | CS | Remiss. |
| 9 | [17] | 3 | 14 | 2.7 | 1.9 | N | | n.d. | CS,CP | Remiss. |
| 10 | [18] | 3 | 7 | 6 | n.s. | N | | DGS+MCD | CS,CB | Remiss. |
| 11 | [18] | 2+7 mo | 2 | n.s. | n.s. | N | | DGS * | CS | CRF |
| 12 | [19] | 9 | 3 | 3.5 | 2.4 | N | | MCD | CP,CS | Remiss. |
| 13 | [20] | 20 | 20 | 3+ | 1.7 | N | | MCD | Diuretics | Pers. prot |
| 14 | [20] | 13 | 13 | 6.8 | 3 | N | | n.d. | None | Remiss. |
| 15 | [21] | 58 | 62 | 11 | 2.7 | N | | DGS+MCD | CSA | Remiss. |
| 16 | [22] | 3 | 28 | 10.5 | 1.3 | Y | 2.3 | DGS | Diuretics | Remiss. |
| 17 | [23] | 3.3 | 3.8 | 32 | 0.8 | N | | MCD | CSA,CS | Remiss. |
| 18 | [23] | 9 | 15.5 | 38 | 2 | N | | DGS+MCD | CS | Remiss. |
| 19 | [23] | 11 mo | 8 | 9 | 2.4 | N | | n.d. | CS | Remiss. |
| 20 | [23] | 4.3 | 4.3 | n.s. | n.s. | N | | n.d. | CS | Remiss. |
| 21 | [24] | 60 | 61 | 16 | 1.8 | Y | 4.5 | MCD | CS | Remiss. |
| 22 | [25] | 67 | 67 | 17 | 1.5 | Y | 3.0 | DGS+MCD | CS, ACEI | Remiss. |

**Note**: ACEI, angiotensin converting enzyme (ACE) inhibitor; Alb, albumin; ARF, acute renal failure; CB, chlorambucil; CP, cyclophosphamide; Cr, serum creatinine; CRF, chronic renal failure; CS, corticosteroids; CSA, cyclosporine ACEI; DGS, mild diffuse diabetic glomerulosclerosis; DM, diabetes mellitus; MCD, minimal change disease; mo, months; N, no; n.d., not done; n.s., not stated; NS, nephrotic syndrome; pers. prot, persistent proteinuria; remiss., remission; UP, urine protein; Y, yes; * biopsy performed 18 years after onset of DM and NS.

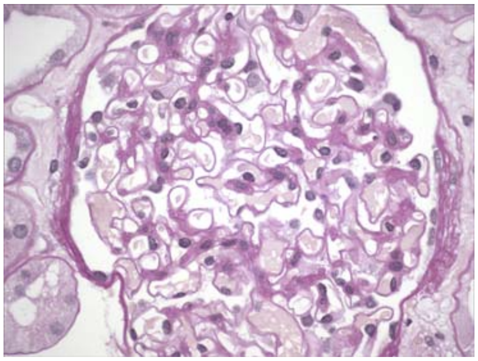

**FIGURE 2.** Renal biopsy findings in a patient with diabetes mellitus and MCD. Glomeruli demonstrate patent capillary lumina and very mild segmental mesangial sclerosis, consistent with early diabetic nephropathy. (Periodic-acid Schiff stain, original magnification × 600.)

mechanisms leading to intravascular volume depletion, renal hypoperfusion, consequent ischemic tubular injury, and interstitial edema[28]. Five of the reported cases of diabetes with reversible nephrotic syndrome also had acute renal insufficiency on initial presentation that improved as the nephrotic syndrome remitted, following steroid therapy in three patients[14,24,25] and spontaneously in the other two[15,22], consistent with renal failure secondary to anasarca.

In summary, MCD should be considered in the differential diagnosis of diabetic patients presenting with sudden onset of massive nephrotic syndrome and acute renal failure, especially in those with no history of pre-existing renal disease. Renal biopsy has an important role in distinguishing diabetic nephropathy from MCD. This differential diagnosis has important therapeutic and prognostic implications, as MCD responds dramatically to steroid therapy whereas diabetic nephropathy is managed by control of hyperglycemia and hypertension. When MCD occurs superimposed on early diabetic glomerulosclerosis, an assessment of the extent of foot process effacement relative to the degree of glomerulosclerosis is an important clue to the correct diagnosis.
